# Assessing regulatory features of the current transcriptional network of *Saccharomyces cerevisiae*

**DOI:** 10.1038/s41598-020-74043-7

**Published:** 2020-10-20

**Authors:** Pedro T. Monteiro, Tiago Pedreira, Monica Galocha, Miguel C. Teixeira, Claudine Chaouiya

**Affiliations:** 1 Department of Computer Science and Engineering, Instituto Superior Técnico (IST), Universidade de Lisboa, Lisbon, Portugal; 2 Instituto de Engenharia de Sistemas e Computadores, Investigação e Desenvolvimento (INESC-ID), Lisbon, Portugal; 3 Instituto Gulbenkian de Ciência (IGC), Oeiras, Portugal; 4 Department of Bioengineering, Instituto Superior Técnico (IST), Universidade de Lisboa, Lisbon, Portugal; 5 iBB - Institute for BioEngineering and Biosciences, IST, Lisbon, Portugal; 6 Aix-Marseille Université, CNRS, Centrale Marseille, I2M, Marseille, France

**Keywords:** Gene regulatory networks, Mathematics and computing

## Abstract

The capacity of living cells to adapt to different environmental, sometimes adverse, conditions is achieved through differential gene expression, which in turn is controlled by a highly complex transcriptional network. We recovered the full network of transcriptional regulatory associations currently known for *Saccharomyces cerevisiae*, as gathered in the latest release of the YEASTRACT database. We assessed topological features of this network filtered by the kind of supporting evidence and of previously published networks. It appears that in-degree distribution, as well as motif enrichment evolve as the yeast transcriptional network is being completed. Overall, our analyses challenged some results previously published and confirmed others. These analyses further pointed towards the paucity of experimental evidence to support theories and, more generally, towards the partial knowledge of the complete network.

## Introduction

Transcriptional regulation is a key mechanism for the control of genomic expression, crucial for the ability of living cells to adapt to environmental stimuli, to thrive under expectable stress and sometimes also under unexpectedly harsh conditions. Despite decades of research in the field, the actual degree of complexity underlying global transcriptional control is yet to be understood. Throughout the years, structural and biochemical studies have highlighted key features of interactions between different specific transcription factors (TFs), and of the role of promoter/enhancer structures in combinatorial regulation of eukaryotic genes^1–3^. There is a consensual opinion that the dominant mechanism underlying the observed complexity of gene expression patterns relies on combinatorial regulation of gene expression^4,5^, defined as the regulation of a single gene by two or more TFs that might either act simultaneously or independently upon different spatial or temporal conditions^6,7^. Nevertheless, general principles underlining the activity of TFs and the combinatorial regulation at a genomic scale are still to be established.

Few, if any, eukaryotic organisms have been as thoroughly investigated as the model yeast *Saccharomyces cerevisiae*. This is particularly true when referring to the study of global transcriptional control, as this organism has been among the first for which extensive analyses of transcription factor activities have been conducted. This effort is illustrated by the analysis of the effect of DNA binding activity for more than one hundred TFs, using Chromatin Immunoprecipitation (ChIP)-on-chip experiments^8^, or the analysis of the effect of the overexpression of 55 TFs in transcriptome remodelling^9^. Moreover, thousands of papers describe transcriptional associations in the model yeast *S. cerevisiae*, obtained through various experimental platforms and observed in many different environmental conditions. The YEASTRACT database, first launched in 2006^10^, and regularly updated since then^11–15^, has provided access to all available data on transcriptional associations in *S. cerevisiae*, gathered from more than a thousand publications in peer-reviewed international journals, and curated by a dedicated team of yeast researchers. YEASTRACT thus provides a great platform to address transcriptional regulation at a global scale.

This accumulation of data on the yeast network has led to several computational studies of topological properties with the intent to uncover design principles of genome-wide transcriptional regulation. Most interest has been focused on in- and out-degree distributions, which correspond to the number of TFs per gene and to the number of target genes (TGs) per TF, respectively^8,16–18^. In 2006, Balaji et al.^17^ assembled and analysed the largest *S. cerevisiae* transcriptional network at that time, relying on the TF-DNA binding evidence data available then.

A network feature that has attracted attention is the over-representation, in the global regulatory network, of small network architectures, referred to as *network motifs*. In this respect, Lee et al. searched for the existence of 6 types of regulatory motifs (auto-regulation, multi-component loop, feedforward loop, single and multi-input motifs, and regulatory chain) in the yeast network^8^, whereas Milo et al. assessed the profiles of significant triads (motifs composed of 3 nodes), i.e., their frequencies in the yeast network compared to randomized networks^19^.

In this paper, we recovered the full network of transcriptional regulatory associations currently known for *S. cerevisiae*, as gathered in the latest YEASTRACT release^15^. Annotations in YEASTRACT were used to produce subnetworks that encompass associations supported by binding and/or expression evidence. In our analyses, we also included networks considered in previous studies. For all these networks, we assessed their topological characteristics aiming at uncovering properties of the transcriptional control of single genes, of specific biological functions and of the complete genome. The consideration of diverse networks allowed to observe that while some properties were verified by all of them, others clearly differed, suggesting that caution should be exercised when drawing general rules on transcriptional genome-wide networks. Furthermore, functional analyses indicated potential relationships between the connectivity (in- or out-degree) of the genes and their biological functions.

## Results

### Transcriptional regulatory networks of *S*accharomyces cerevisiae

There are more than 195,000 regulatory associations between transcription factors (TFs) and target genes (TGs) in *Saccharomyces cerevisiae*, according to the data deposited in the YEASTRACT database^15^, which is the most comprehensive source of such interactions. In YEASTRACT, 1580 references support the stored regulatory associations. Each reference documents an average of 199.68 interactions (0.1% of the total number of interactions, with a standard deviation of 1.29). Interactions are supported by an average of 1.61 references. Table 1 provides statistics for the references that support more than 5% of the YEASTRACT interactions with the total number of documented interactions and the number of interactions supported by another reference.

**Table 1:** Statistics for the five reference documents documenting more than 5% of the 195,470 interactions stored in YEASTRACT.

Number of interactions supported by	Salin et al.^20^	Reimand et al.^21^	Moxley et al.^22^	Chua et al.^9^	Harbison et al.^23^
That ref.	74,131	58,089	25,142	17,756	10,026
(% of YEASTRACT interactions)	(37.92%)	(29.71%)	(12.86%)	(9.08%)	(5.13%)
No other ref.	1966	48,385	20,614	12,294	3955
At least another ref.	72,165	9704	4528	5462	6071

For each reference, the number of documented interactions (and percentage of the total number of interactions in YEASTRACT), the number of those interactions not documented by other references, and the number of interactions documented by at least another reference, are provided.

Regulatory associations in YEASTRACT have been registered based on numerous experimental setups that may be classified into two major groups: (1) those leading to DNA binding evidence, including ChIP-on-chip, ChIP-seq, Electrophoretic Mobility Shift Assay (EMSA), or DNA footprinting; and (2) those leading to expression evidence, which is the demonstration that the deletion, mutation or overexpression of a TF affects the expression of a TG, typically including DNA microarray hybridisation, RNA-sequencing, qRT-PCR, northern blotting or the use of reporter genes. In the sequel, we will denote by:

- *E* the set of regulatory associations supported by expression evidence;
- *B* the set of regulatory associations supported by binding evidence;
- *E*|*B* the set of regulatory associations supported by expression **or** binding evidence (i.e., the whole set of interactions);
- *E*& *B* the set of regulatory associations supported by both expression **and** binding evidence.

Note that the 28 associations in YEASTRACT that still lack annotations of supporting evidence were excluded from the datasets considered in this study.

To assess whether previous observations were maintained in the updated *S. cerevisiae* transcriptional network, we also included in our analyses networks from two reference studies: the Costanzo et al.’s network^24^ that was used by Milo et al. as a source transcriptional network to uncover recurrent network motifs with the intend to disclose topological general principles^19,24,25^; and the Balaji et al.’s network that was assembled to analyse combinatorial regulation on a genomic scale^17^. In both networks, regulatory associations are supported by binding evidence only (indicated as *B*). Note that all the networks considered here are defined over the TFs and genes involved in transcriptional interactions, that is to say, no nodes are isolated.

Table 2 recapitulates the number of nodes and interactions for each of the six networks used in the present work, where, for each network, the nodes are those involved in interactions supported by corresponding evidence. It also indicates the proportion of actual interactions out of the possible interactions between all the identified TFs and genes, showing that the transcriptional networks are rather sparse, with a notable difference between networks containing only interactions supported by binding evidence, and those containing interactions supported by expression evidence.

Intersection assessment between Balaji et al. network on the one hand, and YEASTRACT *B* and *B*&*E* on the other hand shows that: YEASTRACT *B* contains a good proportion of Balaji’s dataset, but around 15% of it has been discarded, whereas YEASTRACT *B*&*E* only contains about 29% of Balaji’s dataset, underlining again the restricted number of associations supported by both binding and expression evidence.

**Table 2:** *(Top)* Statistics of the six *S. cerevisiae* regulatory networks used in this work (TF stands for Transcription Factor, TG for Target Gene).

Regulatory network	Evidence type	# Nodes	# Interactions	# TFs	# TGs	% Possible interactions
Costanzo et al.^24^	*B*	688	1079	131	592	1.20
Balaji et al.^17^	*B*	4441	12,871	159	4408	1.82
YEASTRACT^15^	*B*|*E*	6886	195,470	220	6886	12.90
YEASTRACT^15^	*E*	6711	161,747	215	6711	11.21
YEASTRACT^15^	*B*	6478	45,209	176	6475	3.97
YEASTRACT^15^	*B*&*E*	3937	11,486	152	3912	1.92

Network intersection	# Interactions	% Balaji	% YEASTRACT *B*	% YEASTRACT *B*&*E*		
Balaji $\cap$ YEASTRACT *B*	10,909	84.76	24.26	–		
Balaji $\cap$ YEASTRACT *B*&*E*	3359	26.1	–	29.24		

Each network is defined by a specific dataset, which is related to particular supporting evidence. The last column indicates the proportion of actual interactions out of the possible interactions. For example, for the YEASTRACT *B*&*E* network, there are $152 \times 3937 = 598,424$ such possible interactions, corresponding to the 152 identified TFs regulating all the 3937 TFs and TGs. *(Bottom)* Percentages of interaction overlaps of Balaji et al. with YEASTRACT *B* and with *B*&*E* networks.

Most regulatory associations based on expression evidence are associated with a sign depending on the observed effect on the TG: positive when the TF is an activator, negative when it is a repressor, more rarely dual when it has a dual effect, or possibly unknown when the observed effect has no directionality (i.e., a modified level of expression, but whereas the level increased or decreased was not clear in the supporting article).

**Figure 1:**
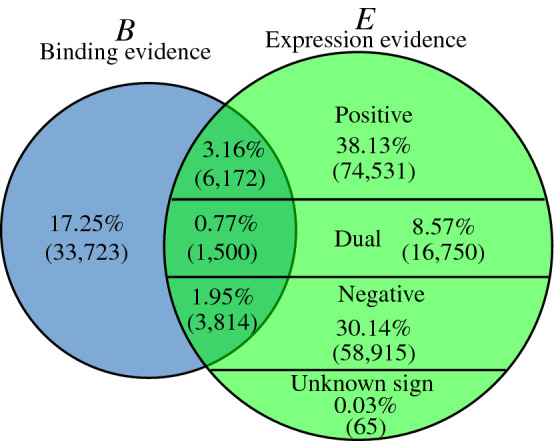
Venn diagram showing the content of YEASTRACT regulatory associations between transcription factors and target genes in *S. cerevisiae* according to their classification in the sets *B*, *E* and *B*&*E*, depending on the supporting experimental evidence. In the set *E* (and *B*&*E*), expression evidence allows to attribute a sign to the associations. Percentages refer to the fractions of the total number of associations, i.e., the cardinality of *B*|*E*.

Figure 1 displays a first assessment of the experimental basis of the data currently available in YEASTRACT. Among the documented regulatory associations, 76.87% are in the set $E\smallsetminus B$, i.e., based on expression evidence only. Part of these associations may be indirect since there might be an intermediate TF through which the registered interaction operates. On the other hand, 17.25% of the documented regulatory associations are based on DNA binding evidence alone (in the set ${B\smallsetminus E}$). This may imply that a TF can bind to a promoter region without affecting the expression of the corresponding gene. If this is the case, it would confirm that binding evidence is not enough to establish a clear regulatory association. This is confirmed by the surprisingly restricted set of regulatory associations that are supported by both DNA binding and expression evidence (i.e., the small cardinality of the set *B*&*E*). Indeed, only 11,486 regulatory associations (5.88% of the whole interaction set *B*|*E*) can be considered reliable with evidence showing that the TF binds to the promoter region of its TG, and promotes or represses its transcription. This small number of associations in *B*&*E* may be explained by the scarcity of expression and DNA binding evidence experiments conducted in the exact same conditions^26,27^. In any case, as those interactions are the most reliable, this analysis suggests that our current knowledge of the transcriptional regulatory network in *S. cerevisiae* is still limited.

### Numbers of regulators and gene functions

#### In-degree analysis

**Figure 2:**
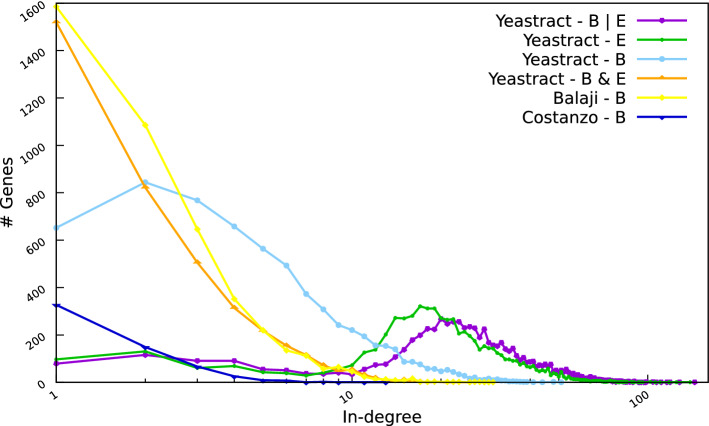
In-degree distributions, i.e., numbers of genes with given in-degrees. Six distinct datasets (or networks) are considered: YEASTRACT *B*|*E* (in purple), YEASTRACT *E* (in green), YEASTRACT *B* (in light blue), YEASTRACT *B*&*E* (in orange), Balaji et al.’s *B*^17^ (in yellow), Costanzo et al.’s *B*^24^ (in dark blue).

In order to assess potential trends in the transcriptional control in *S. cerevisiae*, we first considered the gene in-degrees, i.e., for each gene, the number of TFs controlling its transcription, regarding the different datasets. Plots in Fig. 2 indicate the in-degree distributions for the six versions of the yeast transcriptional network, considering only the regulated genes as in^16,17^. Hence, 3 nodes were excluded for the YEASTRACT *B* network, 25 for the YEASTRACT *B* & *E* network, none for the YEASTRACT *B*|*E* and *E* networks, 33 for the Balaji network, and 96 for the Costanzo network. Supplementary File 3 provides the in-degree distributions without excluding non-regulated nodes, where the four YEASTRACT networks now contain all the nodes present in the E|B network (i.e., the bigger set of genes). These plots further illustrate the lack of regulatory information supported by both binding and expression evidence.

The degree distributions display similar trends for the Costanzo et al., the Balaji et al. and the YEASTRACT *B*&*E* networks, with many genes controlled by a few regulators and a few genes controlled by many regulators. For each of these networks, the degree distribution is a decreasing function. In contrast, when considering the networks from YEASTRACT *B*, *B*|*E* and *E*, the degree distributions tend to bell shaped functions, with reduced numbers of genes having small in-degrees, higher numbers of genes having intermediate in-degrees, and then again reduced numbers of genes having higher in-degrees. It is likely that these distributions follow from a high number of false positive interactions.

The in-degree analysis on the updated YEASTRACT *B*&*E* network thus confirms the previously suggested exponential in-degree distribution of the yeast transcriptional network^16,17^.

#### Relating in-degrees and biological functions

**Figure 3:**
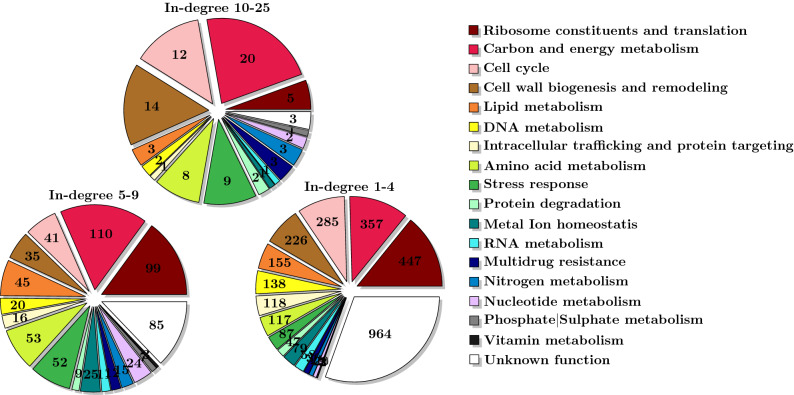
Distribution of biological functions within three in-degree bins, considering the YEASTRACT *B*&*E* network.

To assess whether in-degrees could relate to biological functions, we analysed the distribution of functional categories according to the in-degrees, focusing on interactions in *B*&*E*, i.e., supported by binding and expression evidence. The genes were associated to their major biological functions (see “Methods” section, and Supplementary File 4). To get a better view, we considered bins, gathering genes according to their in-degrees. These bins, appropriately defined (see “Methods” section), are as follows: degrees 1 to 4, 5 to 9, 10 to 25, including 3160, 608 and 144 genes, respectively. The results are displayed in Fig. 3.

In this distribution of functional categories, the genes associated with the *Unknown function* mainly appear in the lower in-degree bin. This is most likely because these genes are less studied. More generally, the vast majority of the genes lies in the bin 1–4.

Considering the genes associated with *Stress response*, 13 (8.78%) of them are in the higher in-degree bin, 87 (58.78%) in the lower 1-4 in-degree bin, and 48 (32.43%) in the intermediate 5–9 in-degree bin. Among the highly regulated stress genes, the small heat shock protein encoding genes *HSP12* (19 regulators) and *HSP26* (12 regulators) are both known to be required in many environmental stress conditions. *HPS12* is activated upon high ethanol concentrations, glucose starvation, cell wall stress, chemical stress, oxidative stress, DNA damage, and plays a role in the protection of protein and lipid folding^28^. Hsp12 is an intrinsically unstructured stress protein that folds upon membrane association and modulates membrane function^29^. Similarly, Hsp26 is activated by a variety of triggers: heat shock, salt shock, cell cycle arrest, nitrogen starvation, carbon starvation, oxidative stress, and low pH^30^.

**Figure 4:**
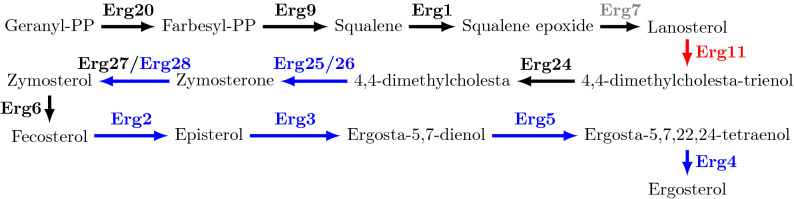
Ergosterol biosynthesis pathway, highlighting the differences in terms of individual gene regulation. Proteins in red, blue or black are encoded by genes whose transcriptional in-degree lays within the 10–25, 5–9 or $<5$ bins, respectively. In grey, ERG7 is not part of the B&E network.

In the functional category *Lipid metabolism*, there are four (1.97%) genes in the higher in-degree bin: FAA1 (15 regulators), OLE1 (14 regulators), ERG11 (11 regulators), and LAC1 (10 regulators). Lac1 is a ceramide synthase subunit, that plays a crucial role in sphingolipid biosynthesis. Indeed, it catalyses the enzymatic branch point in sphingolipid biosynthesis, joining a sphingosine backbone to a very long fatty acid chain, giving rise to ceramide and controling ER-to-golgi traficking of GPI-anchored proteins. Faa1 is one of four fatty acyl-CoA synthetases, but on its own accounts for most acyl-CoA synthetase activity in yeast cells, playing a central role in both glycerolipid and sphingolipid metabolism. Ole1 is the single fatty acid desaturase in *S. cerevisiae*, required for mono-unsaturated fatty acid synthesis. Unsaturated fatty acids are essential for all eukaryotes as key components of cellular membranes that control the membrane organisation, fluidity and permeability, especially under stress. Finally, Erg11 is a lanosterol 14-alpha-demethylase responsible for a key step of ergosterol biosynthesis. Its inactivation not only blocks ergosterol biosynthesis, but it also leads to the accumulation of a toxic ergosterol intermediate. Other ergosterol biosynthesis encoding genes with intermediate in-degrees include *ERG2*, *ERG3*, *ERG4*, *ERG5*, *ERG25*, *ERG26*, *ERG28*, while the remaining steps of the pathway are regulated by a relatively low number of TFs, suggesting that they are not bottlenecks in ergosterol biosynthesis (Fig. 4).

Finally, in the case of the *Multidrug resistance* (MDR) functional category, only three genes (6.67%) belong to the 10–25 in-degree bin: *TPO4* with 12 regulators, and *AQR1* and *PDR5* with both 11 regulators. These genes are involved in multiple functions^31,32^. In particular, *PDR5*, controls resistance to a wide range of unrelated drugs, steroid transport, response to cation stress and cellular detoxification even when growing exponentially in liquid culture^31^. However, most MDR genes (66.67%) have less than five regulators. This is notably the case for genes encoding MDR transporters of the ATP-Binding Cassette (ABC), *PDR12*, *PDR15* and *YOR1*, and of the Major Facilitator Superfamily (MFS) *AZR1*, *DTR1*, *HOL1*, *QDR1*, and *QDR3*, as well as the MDR transcription factors encoded by *PDR1* and *PDR3*. Some of these genes have very specific functions. For example, although Pdr12 belongs to the ABC drug efflux pump family, it has been shown to have a distinct role in the transport of weak organic acids of intermediate lipophilicity such as sorbic and benzoic acids^33^. Another interesting example is the case of Pdr1, the major regulator of MDR in yeast, whose activation occurs by direct binding to drug molecules and not by increased expression in the presence of drugs^34^.

### Numbers of targets and functions of transcription factors

#### Out-degree analysis

**Figure 5:**
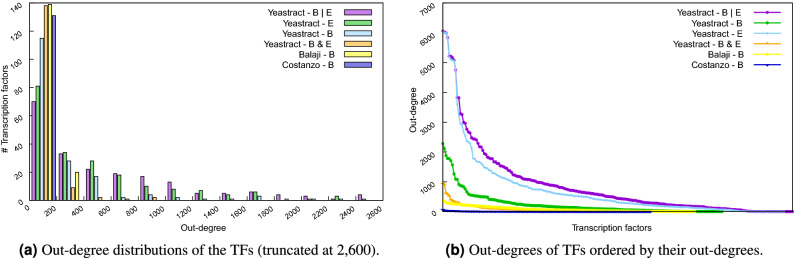
Six distinct datasets (or networks) are considered: YEASTRACT *B*|*E* (purple), YEASTRACT *E* (green), YEASTRACT *B* (light blue), YEASTRACT *B*&*E* (orange), Balaji et al.’s *B*^17^ (yellow), Costanzo et al.’s *B*^24^ (in dark blue). *(Left)* TF out-degree distributions, i.e., number of TFs with each range of number of TGs (less than 200, between 200 and 400, etc.), truncated at 2600. *(Right)* TF out-degree values, in decreasing order.

Here, we considered TF out-degrees, in order to evaluate the distribution of the number of controlled genes per TF (Fig. 5). This analysis was performed on the six networks presented in Table 2. The out-degree distribution is a decreasing function, following the same trend for all the networks, contrary to the behaviour of the in-degree distributions (Figs. 2 and 5). These results are in line with previous observations of a power law out-degree distribution in the *S. cerevisiae* transcriptional network^16–18^.

#### Relating out-degrees and biological functions

To evaluate if out-degree values could have a functional meaning, we focused on the YEASTRACT *B*&*E* network, which includes more reliable associations, and we assessed the functions of the 25 TFs with higher out-degrees and the 25 TFs with lower out-degrees.

Identified TFs with lower and higher out-degrees were grouped according to their major functional categories (Table 3). Overall, this analysis suggests that a few functions are specific to TFs with high out-degrees and others are specific to TFs with low out-degrees. This is the case for the function *Ubiquitous*, which is associated to TFs playing a role in the response to, and control of, a broad range of biological processes (such TFs are impossible to assign to a specific biological process, and appear to act more like general TFs). This function associated to the TFs Cbf1, Rap1, Sfp1, Ifh1 and Fhl1, stands out as exclusive of this higher out-degree group, that is no TF with a degree lower than 3 is associated with this function. Significantly, these transcription factors are responsible for the simultaneous transcriptional control of genes involved in numerous cell functions, including cell cycle, carbon and nitrogen metabolism, or ribosome biogenesis.

The functional categories *Alternative carbon sources*, *Sulphite metabolism* and *Unknown function* appear at the other extreme (out-degrees lower or equal than 3), and are not associated with TFs with higher out-degrees. TFs in the *Unknown function* thus seem to control a small number of genes that are not well characterised. The *Alternative carbon sources* function includes transcription factors such as Azf1, Gal80, Aca1, Mal63 or Usv1, which control the use of non-preferential carbon sources including galactose, maltose, sucrose, oleate, glycerol, acetate or ethanol. These factors control a limited number of genes as it takes only a few metabolic steps to convert the alternative carbon sources into a metabolite that can then be channelled through central carbon metabolism pathways such as glycolysis/gluconeogenesis and the TCA cycle. The function *Sulphite metabolism* includes a single factor, Fzf1, and involves a relatively low number of genes, acting directly on the uptake and metabolisation of sulphur containing molecules.

**Table 3:** The set of 25 TFs with higher out-degree (higher than 91) and 25 TFs with lower out-degree (lower or equal than 3) from the YEASTRACT *B*&*E* network.

Higher out-degree			Lower out-degree		
TF	# Targets	Function	TF	# Targets	Function
Cbf1	622	Ubiquitous	Stb4	3	Unknown function
Rap1	573		Yjl206c	1	
Fhl1	199		Hms2	1	
Sfp1	187		Ppr1	3	Nucleotide metabolism
Ifh1	126		Urc2	2	
Ino4	183	Lipid metabolism	Gzf3	3	Nitrogen metabolism
Ino2	172		Fzf1	2	Sulphite metabolism
Ixr1	202	Hypoxia	Cha4	2	Aminoacid metabolism
Gcn4	517	Aminoacid metabolism	Azf1	2	Alternative carbon source
Met32	336		Gal80	2	
Msn2	905	Stress response	Rsf2	2	
Msn4	395		Mig2	2	
Yap1	297		Aca1	1	
Yrr1	220		Mal63	1	
Cin5	203		Rtg2	1	
Rpn4	181		Usv1	1	
Pdr1	172		Rds1	3	Stress response
Skn7	152		Yap3	2	
Hsf1	138		Haa1	2	
Ste12	942	Cell cycle	Rdr1	1	
Sok2	364		Ime1	3	Cell cycle
Tec1	311		Kar4	3	
Fkh1	290		Ndd1	2	
Swi4	175		Tbf1	2	
Ndt80	169		Wtm1	1	

While the remaining functional categories include both TFs with high and low out-degrees, these TFs play different role in these processes; a factor with a higher out-degree displays a major influence on the associated processes. For example, transcriptional regulators involved in the *Stress response* include those required for the so-called general stress response, Msn2 and Msn4, which control the expression of many genes in response to a multitude of stress stimuli, including heat shock, osmotic shock, oxidative stress, low pH, glucose starvation, sorbic acid and high ethanol concentrations^35^. The major regulators of oxidative stress response in yeast, Yap1 and Skn7, are also present in the 25 TFs with higher out-degrees. The regulatory network underlying oxidative stress response is known to be highly complex, encompassing a large number of genes, as oxidative stress leads to damage in most of the cell components.

Remarkably, the *Stress response* category is also well represented among the 25 TFs with lower out-degrees. However, these TFs have a more restricted role in each process, as they target only a few genes involved in the response to stress agents for which the cell displays more specific and narrow-scope response mechanisms.

Of course, we cannot exclude the possibility that the low out-degree of some of these TFs (e.g. Haa1) also reflects the lack of expression and/or DNA binding experiments conducted in the precise conditions in which they are activated.

### Enriched regulatory motifs in the *S. cerevisiae* transcriptional network

**Figure 6:**
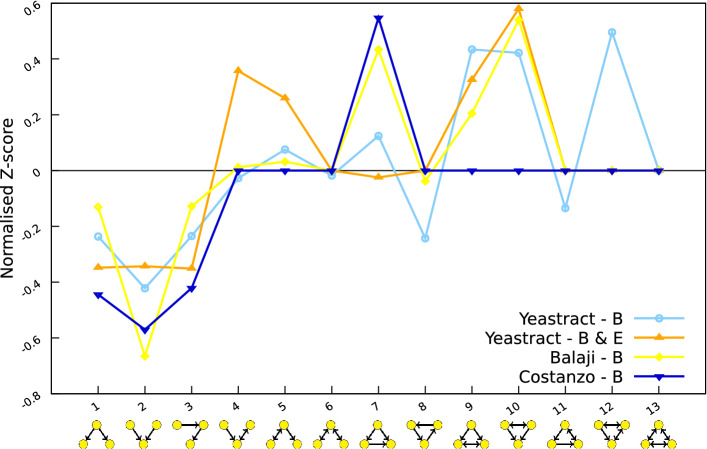
Motif profiles of four *S. cerevisiae* transcriptional networks.

In 2004, Milo et al. presented an approach to assess network local structures in terms of recurring transcriptional motifs. In particular, they considered the *triad significance profile*, which relates to the enrichment significance of the numbers of directly connected triads (defined as 3 node subgraphs) in a given network, compared to these numbers in random networks. Here, we repeated this analysis to assess the motif profile of the YEASTRACT database content, restricted to triadic motifs. We compared the enrichment of the 13 triadic motifs for the datasets from Costanzo et al.^24^ (used by Milo et al.^19^ in their analysis), from Balaji et al.^17^ and from the YEASTRACT binding (*B*) and binding and expression (*B*&*E*) evidence (Fig. 6).

According to the analysis performed by Milo et al.^19^, biological networks would exhibit a motif profile that is preserved across organisms, with a clear under-representation of the triads 1, 2 and 3, and over-representation of the motif 7, known as *feed-forward loop* (dark blue profile in Fig. 6). Interestingly, this profile is not conserved when considering other versions of the yeast transcriptional network (Fig. 6). In particular, the feed-forward loop is less over-represented in the YEASTRACT networks, and the motif 10 is over-represented in all but Costanzo et al. network. In this triad, two TFs cross-regulate themselves and target the same gene. Altogether, these results suggest that the over-representation of specific motifs depends on the data underlying the considered transcriptional network.

The regulatory associations documented in the YEASTRACT datasets relate to all experimental conditions, whereas the networks considered by Milo et al. and that of Balaji et al. are based on data obtained for cells growing under *Control* conditions. In order to analyse comparable datasets, we isolated the YEASTRACT interactions seen to occur only in *Control* conditions, confirming the different motif profiles (Supplementary File 5), with the exception of the motif 12, which is not over-represented anymore. Indeed, different environmental conditions may lead to the activation of different TFs and potentially to different motif profiles. To test this hypothesis, the YEASTRACT network of transcriptional associations supported by binding and expression evidence (*B*& *E*) was filtered according to the specific environmental conditions in which the associations were found to take place. We performed this analysis for the two transcriptional networks as stored in YEASTRACT releases of 2017^14^ and 2019^15^ (the Supplementary File 6 provides an overview of the differences between these two networks). We observed that the motif profiles for the *Control* environmental condition of the 2017 and 2019 networks are similar, whereas they differ for the *Stress* environmental condition (see Supplementary File 7). This highlights again that motif profiles may vary as new data become available (even with small variations as it is the case here). It also supports the fact that there is no immediate functional significance of the over-representation of a network motif^36^, and that function does not follow motif structure neither structure follows function, as proposed by Payne and Wagner^37^.

Altogether these results show that, unless having the “full network”, one should not draw definite conclusions on motif profiles, as these vary significantly between different versions of the transcriptional network.

## Discussion

Networks have proved to be a convenient unifying representation for a wide range of biological processes involving genes, proteins, metabolites, etc. Indeed, cellular responses to environmental signals are governed by complex networks encompassing protein–protein, protein-to-gene and metabolic interactions. In this work, the transcriptional regulatory network of the model yeast *Saccharomyces cerevisiae* was globally evaluated in terms of topological features, using previous data as well as the most recent data available in the YEASTRACT database.

Investigation of the regulatory associations gathered into YEASTRACT database highlighted the lack of associations supported by binding evidence. This suggests that the current network of regulatory associations in *S. cerevisiae* is far from being complete (Fig. 1). Further systematic assessment of transcriptional regulation in yeast is thus still needed, combining different technical approaches.

An exponential distribution of the in-degree in the yeast network was previously suggested, where a few genes would be regulated by a high number of regulators, whereas many genes would be regulated by a few regulators^16,17^. Our analyses showed that, while this distribution seems to be maintained in the YEASTRACT *B*&*E* network, this is not the case when considering larger, yet less reliable YEASTRACT networks (Fig. 2).

By relating functions and in-degrees, it appeared that, given specific biological processes, some involved genes are more tightly regulated than others, operating in broader scopes of conditions. This is the case for the stress responsive genes *HSP12* and *HSP26* that have over 12 regulators, and that are involved in a variety of stress conditions. In contrast, a good proportion (58.78%) of the stress genes have less than 5 regulators, and seem to be activated under the control of a lower number of signaling pathways. Furthermore, we found that for the *Lipid metabolism* functional category, most genes have less than 10 regulators, except a few genes that seem to require a tighter regulation because of their critical role. For example, the crucial nature of the step catalysed by Erg11 in ergosterol biosynthesis might explain why *ERG11* has a higher in-degree, when compared to other players in lipid metabolism. Furthermore, the importance of ergosterol in the organisation of the plasma membrane may explain the intermediate in-degree of a good part of the remaining genes controlling its production. Finally, in the case of the *Multidrug resistance* genes, most of them have a low in-degree. A possible explanation for this may be that yeast does not often resort to the MDR network since natural habitats are normally devoided of chemical stress agents such as drugs. Nevertheless, conclusions about genes with lower in-degrees should be taken with caution. Indeed, a low number of regulators may be due to the lack of knowledge, as suggested by the high number of such genes associated with *Unknown function*.

Our analysis of TFs out-degrees confirmed the power law distribution previously proposed by different authors. It also corroborated the property established by Ouma et al.^18^, basically stating that subnetworks resulting from a sampling of the associations (i.e., the edges) would follow a power law out-degree distribution. According to what was observed for the in-degrees, the TFs with high out-degrees are known to be key regulators involved in a range of biological processes, whereas the TFs with low out-degrees would play a role in very specific functions or have yet no known function.

To uncover potential structural design principles of the yeast transcriptional network, we performed an analysis of motif profiles, focusing on triads. We showed that these motif profiles vary as the network is enriched with novel regulatory associations, and that they also depend on the environmental conditions in which the regulatory associations take place.

Altogether, our study provides a detailed analysis of the most recent version of the transcriptional network, as stored in the YEASTRACT database. Moreover, it highlights the complexity of the transcription regulatory processes that control gene expression, as well as our limited knowledge of the complete *S. cerevisiae* regulatory network. While previously observed properties were confirmed, other could not be retrieved when considering the up-to-date *S. cerevisiae* network. Hence, it seems to be hard to find general principles concerning the yeast transcriptional network as long as the complete network is not known. Still, while being cautious in trying to get definite conclusions, assessment of current genome wide transcriptional networks from topological and functional view points can still be informative. Promises of network biology probably rely on disclosing biological mechanisms rather than general principles.

## Methods

All networks considered (Table 2), as well as processing scripts and intermediate results mentioned below are provided as Supplementary Files.

### Data extraction from the YEASTRACT database

The YEASTRACT database contains all regulatory associations between transcription factors and target genes documented in *Saccharomyces cerevisiae*, up to July 2019. These regulatory associations were obtained from thousands of publications and curated by experts^15^.

The full network (*B*|*E*) considered is provided in Supplementary File 1. Regulatory associations from the full network are annotated with information extracted from one or more supporting papers, whenever such information is available. Regulatory associations can be classified according to the set of employed experiments as: *Binding*, when having at least one publication based on TF–DNA binding assays; or *Expression*, when having at least one publication based on expression evidence and no publication based on TF–DNA binding assays. Then, regulatory associations having at least one publication based on expression evidence, are further classified as: *Positive*, when all publications supported a positive effect; *Negative*, when all publications supported a negative effect; or *Dual*, otherwise.

Additionally, each regulatory association is annotated with the environmental condition in which it was found to take place, such as: Biofilm formation, Carbon source quality/availability, Cell cycle/morphology, Human niche conditions, In vitro, Lipid supplementation, Nitrogen source quality/availability, Oxygen availability, Stress, Unstressed log-phase growth (control).

Supplementary File 2, provides a script with trimming capabilities to generate all the sub-networks and associated network measures from Table 2 derived from the original full network. For example, networks used for the motif profile analyses displayed in Supplementary Files 5 and 7 include regulatory associations supported by both *Binding* and *Expression* evidence, where the full network is further filtered by a given environmental condition.

### Node degree analysis

Supplementary File 2, provides the degree.py Python script to compute, for each network in Table 2, the in- and out-degrees of all the network’s nodes, saving these results in intermediate files. The script then uses these intermediate files to call GNUplot in order to generate the in- and out-degree distributions comparing all networks. In particular, it generates Figs. 2 (for the in-degree) and 5 (for the out-degree). Additionally, it selects the 25 TFs with higher and the 25 TFs with lower out-degrees used in Fig. 3.

### Functional analysis

In order to evaluate a possible link between gene in-degree or TF out-degree and the functional categories associated to these genes and TFs, we performed a classification of the biological function of all the genes/TFs in the YEASTRACT *B* & *E* network, based on the description available in the Saccharomyces Genome Database (http://www.yeastgenome.org), as well as the associated Gene Ontology terms and relevant literature. This classification was manually performed for genes/TFs with at least 5 regulators (752 genes), which were then used to train a Support Vector Machine text classifier based on the Python scikit-learn (https://scikit-learn.org/) library. We then used this text classifier to classify the remaining 3,160 genes. The code is available in Supplementary File 2.

To obtain statistically significant groups, we performed Chi-squared tests for association between consecutive in-degrees, which led to the definition of three bins: in-degrees 1–4, 5–9 and 10–25 (see Supplementary File 4).

The number of genes associated with each biological function in each of the three bins is presented in Fig. 3. Additionally, the 25 TFs with higher out-degree and the 25 TFs with lower out-degree are presented in Table 3 together with their functional classifications. Supplementary File 4 includes the Spreadsheets for both the in-degree and out-degree functional classifications.

### Motif analysis

In order to assess the network recurring transcriptional motifs, we considered the *triad significance profile* presented by Milo et al.^19^, where the number of connected triads in the network is computed and compared with random networks of the same dimensions in order to assess each triad enrichment significance (Z-score). To compute the triad enrichment, we considered the motif discovery software tool gtrieScanner (http://www.dcc.fc.up.pt/gtries/) by Pedro Ribeiro^38^.

Supplementary File 2 provides the motifs.py Python script which, for each network from Table 2, starts by converting the supplied network of genes into an equivalent network of numerical nodes. It then computes the triad enrichment, using gtrieScanner with the following parameters: -s 3 for motifs of size 3, -m subgraphs dir3.str for the subgraph list of size 3 to be considered, -d to consider directed graphs, -f simple for non-weighted graphs, -r 10000 to generate 10,000 random networks and -g network to supply the numerical network previously created. It produces the result file containing the corresponding Z-score for each motif, and a file listing all occurrences of the motifs in the supplied numerical network. Then, the motifs.py Python script parses the results and automatically performs the necessary corrections considered by Milo et al.^19^: (a) the Z-score of a given motif is set to 0 if the motif has less than 4 occurrences; (b) the *triad significance profile* is computed as normalisation of the Z-score to length 1 considering the following formula: $SP_i = Z_i / (\sum_{j=1}^n Z_j^2)^{1/2}$. The normalised *triad significance profiles* of all networks are saved in corresponding files which are used by GNUplot to produce the motif profiles in Fig. 6 and Supplementary Files 5 and 7.

## Supplementary information

Supplementary Information 1.

Supplementary Information 2.

Supplementary Information 3.

Supplementary Information 4.

Supplementary Information 5.

Supplementary Information 6.

Supplementary Information 7.
