# Supplementary material for: Assessing regulatory features of the current transcriptional network of Saccharomyces cerevisiae
Source: Sci Rep. 2020 Oct 20;10:17744. doi: 10.1038/s41598-020-74043-7 (PMC7575604; doi:10.1038/s41598-020-74043-7)

Assessing regulatory features of the current transcriptional network of *Saccharomyces cerevisiae*

Pedro T. Monteiro, Tiago Pedreira, Monica Galocha,
Miguel C. Teixeira, Claudine Chaouiya

Supplementary file 3: In-degree distributions, *i.e.*, numbers of genes with given in-degrees for the YEASTRACT $B|E$ (in purple), YEASTRACT E (in green), YEASTRACT B (in light blue), YEASTRACT $B\&E$ (in orange), where these networks now include the 6,886 nodes present in the $E|B$ network (*i.e.*, the bigger set of genes), and where non-regulated genes (with in-degree 0) are kept.

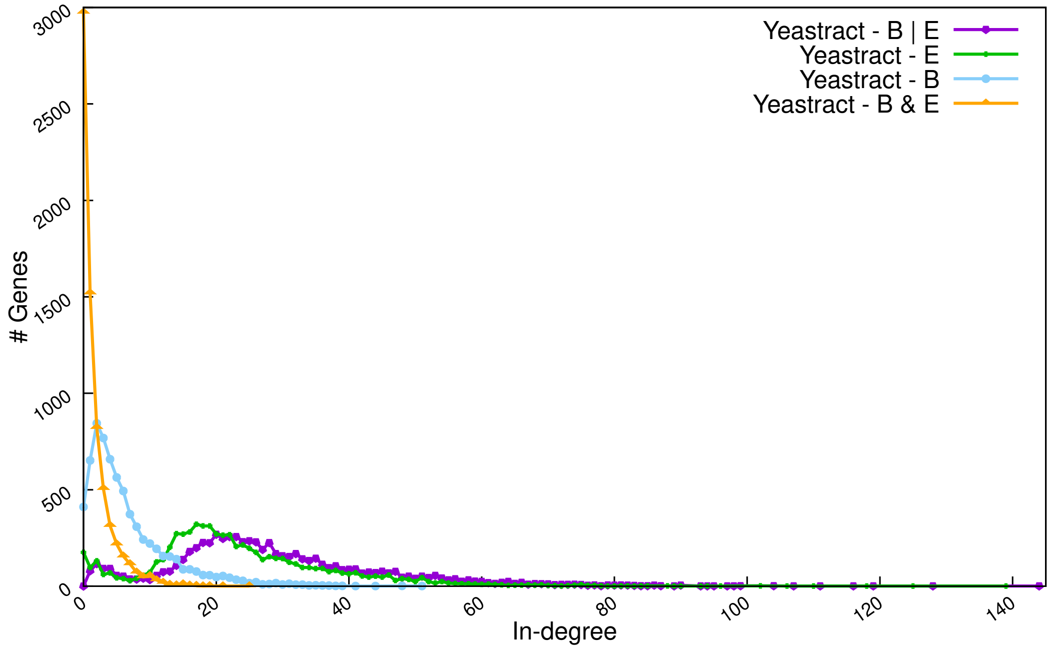

Supplement: Supplementary file 3 — Supplementary Information 3. [file 41598_2020_74043_MOESM3_ESM.pdf]
