# Supplementary material for: Assessing regulatory features of the current transcriptional network of Saccharomyces cerevisiae
Source: Sci Rep. 2020 Oct 20;10:17744. doi: 10.1038/s41598-020-74043-7 (PMC7575604; doi:10.1038/s41598-020-74043-7)

Assessing regulatory features of the current transcriptional network of *Saccharomyces cerevisiae*

Pedro T. Monteiro, Tiago Pedreira, Monica Galocha,
Miguel C. Teixeira, Claudine Chaouiya

Supplementary file 5: Motif profiles of the networks YEASTRACT *B*, YEASTRACT *B&E*, Balaji and Costanzo, considering only regulatory information under *Control* environmental conditions.

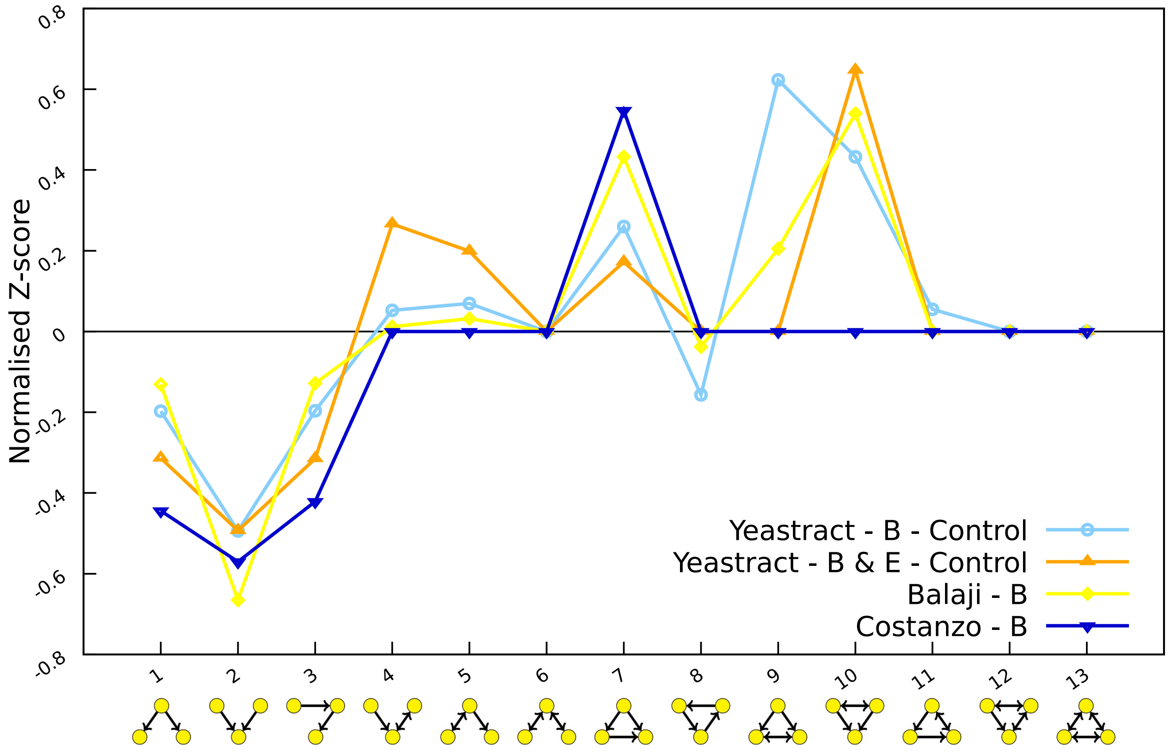

Supplement: Supplementary file 5 — Supplementary Information 5. [file 41598_2020_74043_MOESM5_ESM.pdf]
