# Supplementary material for: Assessing regulatory features of the current transcriptional network of Saccharomyces cerevisiae
Source: Sci Rep. 2020 Oct 20;10:17744. doi: 10.1038/s41598-020-74043-7 (PMC7575604; doi:10.1038/s41598-020-74043-7)
Supplement: Supplementary file 6 — Supplementary Information 6. [file 41598_2020_74043_MOESM6_ESM.pdf]

Assessing regulatory features of the current transcriptional network of *Saccharomyces cerevisiae*

Pedro T. Monteiro, Tiago Pedreira, Monica Galocha,
Miguel C. Teixeira, Claudine Chaouiya

Supplementary file 6: Statistics from the YEASTRACT full networks from 2017 release and from 2019 release, as well as their sub-networks filtered by environmental conditions.

Regulatory network	Evidence type	Env. cond.	# Nodes	# Interactions	# TFs	# TGs
YEASTRACT (2017)	$B\|E$	All	6,853	161,029	207	6,853
YEASTRACT (2017)	E	All	6,604	125,968	200	6,604
YEASTRACT (2017)	B	All	6,545	44,565	175	6,543
YEASTRACT (2017)	$B\&E$	All	3,653	9,504	148	3,622
YEASTRACT (2017)	$B\&E$	Control	3,099	6,894	146	3,055
YEASTRACT (2017)	$B\&E$	Cell Cycle	1,386	1,803	21	1,379
YEASTRACT (2017)	$B\&E$	Carbon	713	948	46	676
YEASTRACT (2017)	$B\&E$	Nitrogen	728	990	47	704
YEASTRACT (2017)	$B\&E$	Stress	1,976	3,225	74	1,942
YEASTRACT (2019)	$B\|E$	All	6,886	195,470	220	6,886
YEASTRACT (2019)	E	All	6,711	161,747	215	6,711
YEASTRACT (2019)	B	All	6,478	45,209	176	6,475
YEASTRACT (2019)	$B\&E$	All	3,937	11,486	152	3,912
YEASTRACT (2019)	$B\&E$	Control	3,490	8,857	149	3,455
YEASTRACT (2019)	$B\&E$	Cell Cycle	1,479	1,951	27	1,471
YEASTRACT (2019)	$B\&E$	Carbon	1,802	3,268	100	1,755
YEASTRACT (2019)	$B\&E$	Nitrogen	975	1,356	52	951
YEASTRACT (2019)	$B\&E$	Stress	2,068	3,576	84	2,029
