# Supplementary material for: Assessing regulatory features of the current transcriptional network of Saccharomyces cerevisiae
Source: Sci Rep. 2020 Oct 20;10:17744. doi: 10.1038/s41598-020-74043-7 (PMC7575604; doi:10.1038/s41598-020-74043-7)

Assessing regulatory features of the current transcriptional network of *Saccharomyces cerevisiae*

Pedro T. Monteiro, Tiago Pedreira, Monica Galocha, Miguel C. Teixeira, Claudine Chaouiya

Supplementary file 7: Motif profiles of the networks YEASTRACT *B&E* from 2017 and YEASTRACT *B&E* from 2019, considering only regulatory associations under the *Stress* and *Control* environmental conditions.

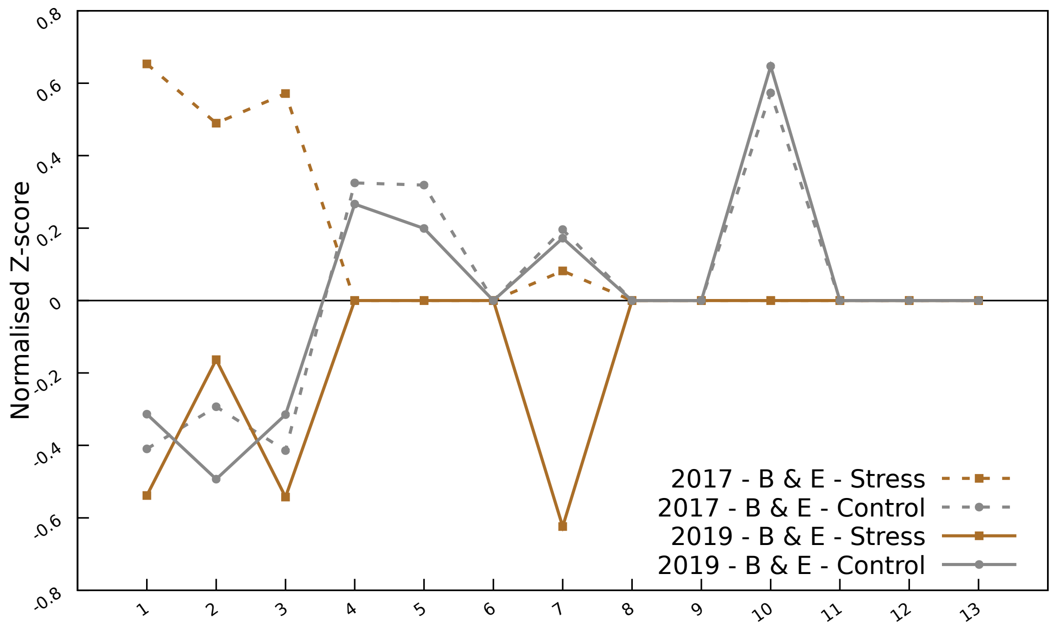

Supplement: Supplementary file 7 — Supplementary Information 7. [file 41598_2020_74043_MOESM7_ESM.pdf]
